# Quality Control of Microscopic Diagnosis of Malaria in Healthcare Facilities and Submicroscopic Infections in Mossendjo, the Department of Niari, the Republic of the Congo

**DOI:** 10.3390/pathogens13080709

**Published:** 2024-08-21

**Authors:** Grâce Petula Urielle Fila-Fila, Felix Koukouikila-Koussounda, Fabien Roch Niama, Lauriate Prudencie Bissombolo Madingou, Jordy Exaucé Demboux, Aldi Fred Mandiangou, Stéphane Vembe Mahounga, Ahmed Jordy Doniama, Louis Régis Dossou-Yovo, Prisca Nadine Casimiro, Pembe Issamou Mayengue

**Affiliations:** 1 Département de Biologie Cellulaire et Moléculaire, Faculté des Sciences et Techniques, Université Marien Ngouabi, Brazzaville P.O. Box 69, Congo; gfilafila@gmail.com (G.P.U.F.-F.); felixkoukouikila@gmail.com (F.K.-K.); fabien.niama@gmail.com (F.R.N.); lauriatemadingou@gmail.com (L.P.B.M.); jordydemboux@gmail.com (J.E.D.); maldifred@gmail.com (A.F.M.); christvembe@yahoo.fr (S.V.M.); jordydoumdoniama@gmail.com (A.J.D.); priscacasimiro137@gmail.com (P.N.C.); 2 Laboratoire National de Santé Publique, Brazzaville P.O. Box 120, Congo; doyorel2@gmail.com; 3 Ecole Normale Supérieure, Université Marien Ngouabi, Brazzaville P.O. Box 69, Congo; 4 Institut National de Recherche en Sciences de l’Ingénieur, Innovation et Technologie, Cité Scientifique de Brazzaville, Route de l’Auberge de Gascogne, Brazzaville P.O. Box 181, Congo

**Keywords:** malaria, diagnosis, microscopy, submicroscopic infection, Mossendjo, Republic of the Congo

## Abstract

The control and management of malaria are linked to the quality of diagnosis. We sought to estimate the performance of routine microscopy for malaria diagnosis and assess the prevalence of submicroscopic *Plasmodium* (*P.*) *falciparum* infection among febrile patients in two healthcare facilities in Mossendjo, the Republic of the Congo. A cross-sectional study was conducted between January and December 2022. A total of 650 and 234 patients with signs of uncomplicated malaria were enrolled at the Centre de Sante Intégré (CSIMSJ) and Hôpital de Base (HBMSJ), respectively. Two thick blood smears were performed for each patient, one analyzed by routine microscopists and the other by an expert. The *msp-1* and *msp-2* genes were genotyped to detect submicroscopic *P. falciparum* infection. At the CSIMSJ, the sensitivity was 49.5% and the specificity was 88.6%. The positive and negative predictive values were 77.7% and 68.7%, respectively. At the HBMSJ, the sensitivity was 32.9% and the specificity was 79.4%. The positive and negative predictive values were 44.8% and 69.5%, respectively. *P. falciparum* was the only species detected by routine microscopists, while experts identified some cases with *P. malariae* and *P. ovale*. The proportion of submicroscopic infections was 35.75%. Children under 5 years old had higher rates of parasitemia. However, submicroscopic infections were more pronounced in the adult group. The performance of routine malaria microscopists at Mossendjo was inaccurate at both sites. With the large proportion of submicroscopic infection, malaria management at Mossendjo requires the improvement of microscopists’ skills and the concomitant use of RDTs.

## 1. Introduction

Malaria continues to claim many victims worldwide, particularly in tropical and subtropical regions. In 2022, there were an estimated 249 million cases of malaria and 608,000 deaths worldwide, with 94% of cases and 95% of deaths occurring in Africa. Nearly 78% of malaria-related deaths occur in children under the age of 5 years [1]. Despite the efforts made in the fight against malaria, it remains a major public health burden, with a higher incidence and mortality rate than before the COVID-19 pandemic. Various measures implemented and widely used in endemic areas have reduced the morbidity and mortality associated with malaria [2]. The weak execution of different interventions during COVID-19 may be a factor contributing to the rebound of malaria. 

The diagnosis of certainty and appropriate treatment are the cornerstones of malaria management and elimination. Malaria diagnosis constitutes an emergency for the prompt support of patients [3]. Thus, the World Health Organization (WHO) recommends a malaria test for all suspected cases using either microscopy or a rapid diagnostic test (RDT) before treatment [4]. However, an inaccurate diagnosis is problematic for patients, who may develop mistrust in the healthcare system when misdiagnosis leads to wrong treatment decisions [5]. Microscopy is the referential technique for malaria diagnosis. It is comparatively cheap and allows for discrimination between plasmodial species. However, the effectiveness of microscopy is intimately linked to the skill and expertise of the laboratory staff (microscopists), well-maintained microscopes, and the use of good-quality reagents [6]. In general, the detection limit of microscopy is estimated to be between 50 and 500 parasites/μL of blood, although an expert microscopist can detect up to 10 parasites/μL [7]. However, the performance of microscopy is limited in the context of low parasitemia and mixed infections and is tedious for population-level surveillance [8]. 

Meanwhile, RDTs, based on the detection of circulating antigens in blood, have enhanced malaria diagnosis by providing rapid point-of-care diagnosis due to their simplicity in use [9]. Nevertheless, health workers should be trained for better interpretation and credibility of RDT results. This can reduce the constraints of unnecessary treatment, thus reducing the effects of drug pressure that may contribute to the selection of resistant parasites [10]. However, RDTs cannot detect parasitemia at the lowest detection limit, which is the parasitemia level commonly observed in low-malaria-transmission settings [11]. In recent years, the increasing use of molecular methods based on polymerase chain reaction (PCR), which are more sensitive than microscopy in many research settings, has uncovered a large reservoir of submicroscopic *P. falciparum* infections [12], revealing the widespread presence of plasmodial infections with very low parasite densities [13]. Because of the high cost of PCR techniques, it is difficult to integrate them as basic diagnostic techniques in resource-limited countries featuring clinical episodes of malaria.

Malaria remains the main reason for visiting health centers in the Republic of the Congo. The National Malaria Control Program (NMCP) estimated that 63% of consultation cases, 20% of hospitalizations, and 9% of deaths are due to malaria [14]. 

Microscopy is the test widely used for diagnosing malaria in most health facilities, while the confirmatory testing rate with RDTs remains relatively low [15,16]. Compared with other African regions, the Republic of the Congo has also followed WHO recommendations such as distributing long-lasting insecticide-treated bed nets, implementing intermittent preventive treatment (IPT), especially in pregnant women, and using artemisinin-based combination therapies (ACTs) for the treatment of uncomplicated malaria, as well as the early diagnosis of cases [3]. However, several studies aimed at evaluating the impacts of these interventions have mainly been carried out in the capital cities, namely, Brazzaville and Pointe-Noire. Recently, different studies conducted in these cities have demonstrated the following: (1) ACTs remain efficient, and no polymorphisms are associated with resistance to ACTs in *P. falciparum* isolates, while those associated with resistance to chloroquine and sulfadoxine–pyrimethamine remain high; (2) while high genetic diversity was found, despite variation in the prevalence of malaria, parasitemia and the multiplicity of *P. falciparum* infection was noticed throughout the year; and (3) the performance of routine malaria microscopy remains inaccurate, with large variations between many healthcare facilities [15,17,18]. Nevertheless, to our knowledge, very few data are available on submicroscopic plasmodial infections in symptomatic populations and the performance of microscopists in other departments of the Republic of the Congo. This study aimed to evaluate the performance of routine microscopy for malaria diagnosis in two healthcare facilities in Mossendjo in the Department of Niari, the Republic of the Congo, and to assess the prevalence of submicroscopic malaria infection in this area. 

## 2. Materials and Methods

### 2.1. The Study Areas

**Figure 1:**
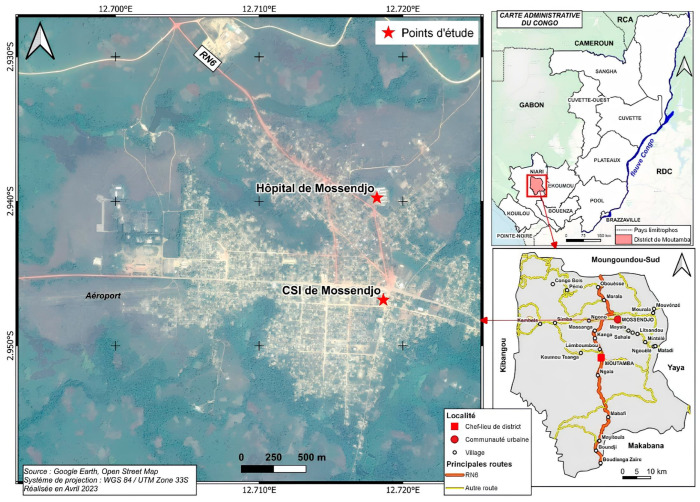
The map representing the region and the different sites of this study.

Mossendjo is the second city of the Niari Department after Dolisie. It is located in the south of the Republic of the Congo, 487 km from Brazzaville, in the heart of the Chaillu massif, full of mountain and forest chains, extending over an area of 5200 km^2^. Mossendjo is a semiurban community with approximately 13,000 inhabitants, mainly living from agricultural activities and hunting. It is divided into two districts. Patient recruitment, blood collections, and thick blood smear examinations were performed at the Centre de Santé Intégré-Poste (CSIMSJ) located in district 1, namely, Bouali, and the Hôpital de Base (HBMSJ) located in district 2, called Itsibou (Figure 1). 

The climate is humid tropical with two rainy seasons observed each year: the main one occurring from February to May, and a short one from October to December [16,19,20]. However, the malaria transmission level in this remote area of the country is not documented. 

The quality control of the thick-film examinations was carried out at the National Public Health Laboratory (LNSP) in Brazzaville. This expert microscopic analysis was performed by two imminent biologists with extensive experience in parasitology working in the field of antimalarial drug resistance testing, who were blinded from the results obtained in the two health centers of this study. Molecular analyses for the detection of submicroscopic infections were conducted at the LNSP.

### 2.2. Ethical Clearance and Site Preparation

This study’s research protocol was approved by the institutional “Comité d’Ethique de la Recherche en Sciences de la Santé” (N°275/MRSIT/IRSSA/CERSSA-2021) of the Republic of the Congo. We obtained the agreement of the Departmental Director of Health and the other territorial administrators to install solar panels at the HBMSJ, providing the necessary electricity to keep blood samples cold and allow the reading of blood smears under a microscope. All the equipment employed, from the collection to the realization of the blood smears, including the microscope, was provided by the project. When the project was launched, we held working sessions with the sites’ actors, including microscopists, allowing them to immerse themselves in the project based on the objectives, the adopted methodology, and the expected results. Simulations were conducted at each site to better address the issue of informed consent via good communication with the patients.

### 2.3. Study Population, Blood Samples, and Data Collection

All patients presenting at one of the study sites with signs of uncomplicated malaria from February 2022 to January 2023 were invited to participate in this study. Those who showed signs of severe malaria or any other pathologies according to the doctor, as well as pregnant women, were excluded. The sampling method used in this study is described in [15]. Briefly, the number samples to be included in this study was estimated by a statistician, considering the proportion malaria cases provided by each health center (given the lack of epidemiological data for the area), one year before the study’s implementation. The sample size was calculated using the Schwartz method, considering a power of 80% and a significance level of 5%, which gave a minimum size of 650 and 234 patients to be recruited at the CSIMSJ and the HBMSJ, respectively. 

Recruited patients were randomly selected from Monday to Friday. At the CSIMSJ, a minimum of 12 patients were recruited per week, with at least 2 patients per day. At the HBMSJ, an average of 4 patients were recruited per week, with 1 per day. After obtaining informed consent from patients or their guardians, investigation sheets were completed by filling in the patients’ information, such as fever or history of fever in the last 48 h, other signs of malaria, origin, previous intake of antimalarial drugs and mosquito net use. Each patient’s axillary temperature was taken for confirmation of fever at each site.

Venous whole blood (2–5 mL) was collected into ethylenediaminetetraacetic acid (EDTA) vacutainer tubes. A small drop of blood was used to prepare thick and thin blood films, and another was collected on filter paper for molecular analyses. The remaining blood was first stored at −20 °C until being transferred to the LNSP and stored at −80 °C for further studies. 

Two blood smears on two different slides were performed according to the WHO protocol for blood smear preparation and subsequent Giemsa staining [6]. The first slide was stained and read at the site to report the patient’s result for possible patient management. The slides were stained with 10% Giemsa for 15 min, gently rinsed with tap water, and then dried before being examined. Each slide was considered negative when approximately 200 microscopic fields were scanned, and no parasite was found. Slides with detected parasites were assessed until 200 leucocytes had been counted. The parasite density was defined as the number of parasites/μL of blood, considering the leukocyte count of 8000/μL of blood per the WHO method. The parasitemia was classified as low (<1000 parasites/μL), moderate (1000–4999 parasites/μL), high (5000–99,999 parasites/μL), or hyperparasitemia (≥100,000 parasites/μL) according to the value ranges [21]. The second slide of each patient was sent to the LNSP for quality control.

### 2.4. Detection of Submicroscopic Infection via Parasite Genotyping 

Genomic DNA was extracted from the filter paper using the zymo Quick kit DNA card (Zymo Research Irvine, CA, USA) according to the manufacturer’s instructions. The molecular detection of *P. falciparum* was performed using the nested PCR technique. Briefly, genes expressing merozoite surface protein-1 (msp-1) and merozoite surface protein-2 (msp-2) were used as polymorphic markers, as described in [22]. The PCR amplification was carried out with a 2-step amplification procedure, including a primary PCR for each gene followed by nested PCRs relating to the different allelic families of each. Positive controls, 3D7 and Dd2, were used for this purpose, and deionized water was considered as a negative control. Five microliters of each of the PCR products was loaded on 2% agarose gel (PeqLab, Erlangen, Germany), stained with ethidium bromide, separated via electrophoresis, and visualized under ultraviolet transillumination [23]. 

### 2.5. Data Analysis

Microsoft Excel software (Microsoft Inc., Redmond, WA, USA), version 2016 was used to record patient data. Graphpad Prism software (Bostoon, New-York, NY, USA), version 9.5 (730) and R 4.3.1 software (Trenton, NJ, USA), version 2023 were used for statistical computing. McNemar’s test was used for quality control, particularly in calculating the sensitivity and specificity of the routine microscopy. The test’s sensitivity was determined as the number of true positives/[number of true positives + number of false positives] × 100. The test’s specificity was determined as the number of true negatives/[number of true negatives + number of false positives] × 100. Positive predictive values (PPVs) and negative predictive values (NPVs) were calculated as described by Ntoumi et al. [16].

A standard error of 5% and a confidence interval set at 95% were used for the different patient characteristics and to evaluate the sensitivity and specificity between the site microscopists and the experts. Kappa values were calculated to assess the level of agreement in the plasmodial species identification between the site microscopists and the experts. The Kappa results were interpreted as follows: values of ≤0 were taken as indicating no agreement, 0.01–0.20 as none–slight, 0.21–0.40 as fair, 0.41–0.60 as moderate, 0.61–0.80 as substantial, and 0.81–1.00 as almost perfect [24].

## 3. Results

### 3.1. Demographic Characteristics of Participants

A total of 884 patients of all ages participated in this study, with 650 and 234 recruited at the CSIMSJ and the HBMSJ, respectively. The patients’ ages ranged from 6 months to 85 years old and from 4 months to 80 years old, with mean ages of 25.96 ± 0.84 years old and 32.01 ± 1.26 years old, at the CSIMSJ and the HBMSJ, respectively. Patients over 15 years old constituted more than half of the study population, with 380 (58.46%) and 193 (82.48%), followed by those between 5 and 14 years old, with 181 (27.85%) and 25 (10.68%), at the CSIMSJ and the HBMSJ, respectively. Children under 5 years old constituted the least-represented group, with 89 (13.69%) and 16 (6.84%), at the CSIMSJ and the HBMSJ, respectively. Women outnumbered men at both sites. Most patients slept under a mosquito net, with 92.92% and 85.90% registered at the CSIMSJ and the HBMSJ, respectively. Patients with fever were estimated at 52.56% and 80.62%, while 27.78% and 6.92% had taken antimalarial treatment a few days before presenting at the HBMSJ and the CSIMSJ, respectively (Table 1).

### 3.2. Quality Control of Malaria Parasite Detection in Health Centers

Out of the 650 blood samples collected at the CSIMSJ, 184 were found to be positive by the site microscopists, of which 143 (22%) were confirmed as positive and 41 (6.31%) were reported as falsely positive by the experts (Table 2). A total of 320 (49.23%) samples were reported as negative after examinations by the expert, while the site microscopists initially reported 466. Thus, 146 (22.46%) samples were considered as false negatives. Of the 234 blood samples collected at the HBMSJ, 58 were reported as positive by the microscopists, of which only 26 (11.11%) were truly positive and 32 (13.67%) were falsely positive after being checked by the experts. Of the 176 samples declared negative by the site microscopists, 123 (52.56%) were reported as negative and 53 (22.65%) as positive by the experts (Table 2).

Thus, the sensitivities were 49.5% (CI: 43.7–55.2%) and 32.9% (CI: 22.5–43.3%), and the specificities were 88.6% (CI: 85.4–91.9%) and 79.4 (CI: 73.2–85.7%) at the CSIMSJ and the HBMSJ, respectively. The positive predictive values were 77.7% (CI: 71.9–82.6%) at the CSIMSJ and 44.8% (CI: 34.3–55.8%) at the HBMSJ. The negative predictive values were 68.7% (CI: 66.0–71.2%) and 69.5% (CI: 66.1–73.4%) at the CSIMSJ and the HBMSJ, respectively. 

### 3.3. Identification of Plasmodial Species

The microscopists at the sites detected only *P. falciparum*. The experts detected two additional species, *P. malariae* and *P. ovale*, in mono- and mix-infections, although *P. falciparum* remained more prevalent, with proportions of 90.31% (261/289) and 88.61% (70/79) at the CSIMSJ and the HBMSJ, respectively.

At the CSIMSJ, 15 samples were positive for *P. malariae* and 8 were positive for *P. ovale*. Four samples showed mixed infections of *P. falciparum* and *P. malariae* and only one had mixed infection of *P. falciparum* and *P. ovale*. At the HBMSJ, only one sample was positive for *P. malariae*, with six positive samples having mixed infections involving *P. falciparum and P. malariae* and two samples being positive for *P. ovale* (Table 3). 

### 3.4. Estimation of Parasitemia at the Health Centers

**Figure 2:**
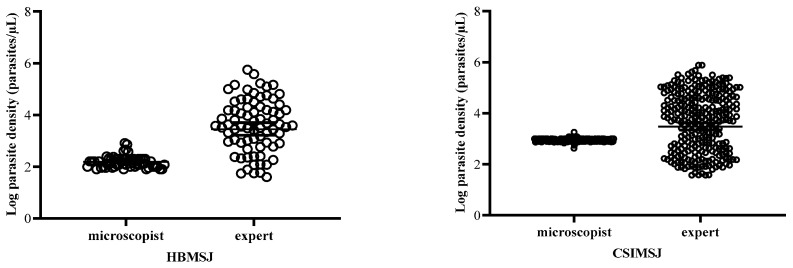
Comparison of parasite density between the sites’ microscopists and the experts.

The geometric means of the parasite densities of the microscopists were 154.08 ± 78.06 and 882.81 ± 110.07, while those obtained by the experts were 4019.79 ± 4251.36 and 4645.50 ± 5562.91 at HBMSJ and CSIMSJ, respectively. Thus, the geometric means of the parasite densities were 5 and 26 times underestimated by the microscopists at the CSIMSJ and the HBMSJ, respectively. Consequently, the microscopists’ estimates were much lower than the experts’ with significant differences (*p* < 0.0001) at both sites (Figure 2).

### 3.5. Prevalence of Microscopic and Submicroscopic Infections in Relation to Age 

**Figure 3:**
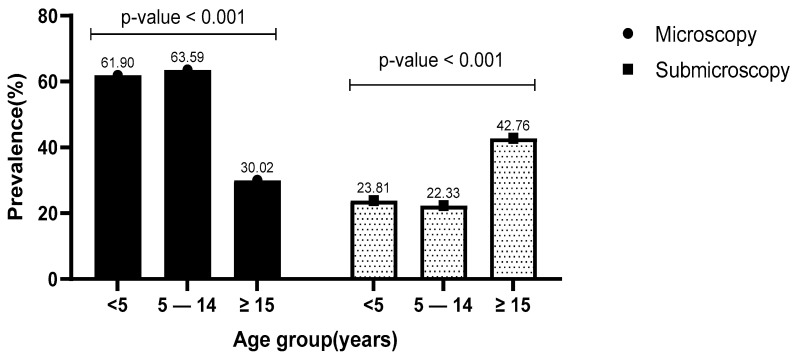
Relationship between microscopic and submicroscopic infections with age.

Considering both sites together, the prevalence of microscopic and submicroscopic infections were 41.63% and 35.75%, respectively (*p* = 0.0076). The prevalence of microscopic infections was statistically higher (*p* < 0.001) in patients aged 5–14 years old (63.59%) than in those under 5 years old (61.90%) or more than 15 years old (22.33%). Inversely, the prevalence of submicroscopic infections was higher in adults (42.76%) than in children under 5 years old (23.81%) and those aged 5–14 years old (22.33%) (*p* = 0.049) (Figure 3). When considering parasitemia in patients with microscopic infection, the mean log10 of parasite density was higher in children under 5 years old (9.41 ± 2.61) and gradually decreased in the age groups of 5–14 years (9.28 ± 2.37) and adult patients ≥ 15 years old (7.34 ± 2.26) (*p* < 0.001).

### 3.6. Relationship of Microscopic Infection, Parasite Density, and Submicroscopic Infections with Demographic and Clinical Variables

The prevalence of microscopic infection was lower in patients who used mosquito nets than in those who did not, at 39.63% and 60%, respectively (*p* < 0.001). The rate of microscopically infected patients was comparable between men and women (*p* = 0.851) and between patients who did and did not take treatment (*p* = 0.917). Nonsignificant differences were observed for parasite density (*p* = 0.128) and submicroscopic infection (*p* = 0.196) between men and women. The average parasite densities were similar in both patients who did and did not use mosquito nets (*p* = 0.898). Likewise, no significant difference was observed in patients who did and did not take antimalarial treatment (*p* = 0.308). However, the prevalence of submicroscopic infections was higher in patients who used mosquito nets than in those who do not, at 37.38% and 21.25%, respectively (*p* = 0.005). Similarly, patients who did not take treatment had a slightly higher prevalence of submicroscopic infections, at 36.62%, compared with the prevalence of 30.91% for those who did (Table 4).

## 4. Discussion

The management and control of malaria require two crucial factors: early diagnosis and adequate treatment. A previous study conducted in Brazzaville in the Republic of the Congo showed that the performance of routine microscopy in different health centers remains substandard [15]. The current study conducted in Mossendjo, a remote area where the malaria situation is still unknown, also confirmed the low performance level of microscopists in diagnosing malaria at the HBMSJ and the CSIMSJ.

The microscopy sensitivity was very low compared with the specificity at both sites. These results are similar to those found in Brazzaville. However, the sensitivity and specificity were significantly higher at one of the considered sites because it was the national site for microscopist training; as such, it had qualified trainers and technicians also working in routine parasitological diagnostics [15]. Similar findings were obtained in Ghana, where the sensitivity (39.3%) was much lower than the specificity (98.2%) [25]. The low sensitivity found in our study can be explained by the high rate of false-negative results, which may be due to microscopists’ inability to identify and distinguish parasites when reading thick blood smears. Since the microscope, reagents, and slides were provided by the project, we believed that the misreading may have been due to the lack of expertise among healthcare workers [7]. Thus, training platforms to enable microscopists to regularly retrain are needed. Generally, misdiagnosis can have considerable repercussions in the delay or failure to treat presumed-negative patients, which could lead to severe forms of the disease or even death.

The false positive rates of 11.4% and 20.6% obtained at the CSIMSJ and HBMSJ, respectively, are similar to the 19% reported in the Democratic Republic of the Congo [26]. However, our results differ from the 2.9% reported in Ethiopia. This result is thought to reflect the tests’ low specificity, with one reason for this being the confusion of parasites with artefacts, which can lead to an overestimation of the diagnosis and, thus, result in unnecessary treatment or a delay in the diagnosis of the actual disease [25,27]. Incorrect identification of parasites may lead to inappropriate treatment and the emergence of parasites resistant to antimalarial drugs [28]. In such a remote area, where training is lacking, the routine diagnosis must be strengthened via the combined use of microscopy and RDT to reduce the occurrence of errors.

*P. falciparum* was the only species identified by the microscopists, while the experts identified mainly *P. falciparum*, followed by *P. malariae* and *P. ovale*. Conversely, the experts in the previous study carried out in Brazzaville identified only *P. falciparum*, while the site microscopists identified at least one *P. malariae* and one *P. ovale*. In addition, the microscopists obtained relatively low parasite density values compared with the experts, as was also observed in studies carried out in the Republic of the Congo and Ethiopia [15,27]. This observation confirms that the microscopists were not sufficiently trained and did not have the necessary experience to estimate parasite densities or distinguish different species of *Plasmodium*. Quantifying or estimating the diagnostic certainty of parasites can be useful for monitoring a patient’s response to treatment and studying drug efficacy [15,27]. Furthermore, the prevalence of *P. malariae* (4.35%) found in this study was slightly higher than that in the surroundings of Brazzaville (1.3%) and Equatorial Guinea (1.4%) [29,30].

A high prevalence of microscopic infections (41.63%) compared with submicroscopic infections (35.75%) was found. The observation contrasts with the findings of Baïna et al., where microscopic infections (33.7%) were less prevalent than submicroscopic infections (66.2%) in the surroundings of Brazzaville [17], as well as in Kenya, with prevalences of 15% and 31% for microscopic infections and submicroscopic infections, respectively [28].

The high prevalence of microscopic infections in Mossendjo may be related to the relief forest, which may influence the malaria endemicity. The submicroscopic infections were also slightly high in this study, and patients with such infections constituted a reservoir for malaria transmission [31,32,33]. Different measures, such as the distribution of long-lasting insecticide-treated mosquito nets, intermittent preventive treatment (IPT), particularly for pregnant women, and treatment with ACTs, have been implemented in Mossendjo. Thus, the high rate of submicroscopic infections could be related to the impact of these measures on the reduction in the *P. falciparum* parasite density [2,3,34].

Children aged between 5 and 14 years old had a higher prevalence of microscopic infections, followed by children under 5 years, and then adults. Similar results were reported in the studies conducted in the surroundings of Brazzaville and the Ashanti Region of Ghana [25,35]. Children aged between 5 and 14 years are more exposed to infectious mosquito bites due to their poor sleeping habits under mosquito nets [36], whereas children under 5 years of age are always mothered and are likely to sleep under mosquito nets with their parents [37]. Conversely, the parasite density was higher in children under 5 years old and progressively decreased with age. This finding is supported by the fact that, being less exposed, their naïve immune systems cannot develop an adapted immune response quickly enough to control the multiplication of many parasites [38,39]. It has been shown that the rate of microscopically detectable plasmodial infections decreases with age and that this may be due to the frequent exposure of individuals to mosquito bites, leading to the acquisition of immunity [40], which helps them suppress the parasite load and leads to an increased prevalence of submicroscopic infections in this age group [41].

Sex and the treatment taken before the consultation did not influence the proportion of microscopic or submicroscopic infections. However, patients who slept under a mosquito net had a higher prevalence of submicroscopic infection than those who did not. We believe that limiting human–vector contact through the use of a net may lower biting and sporozoite inoculation rates, implying that low biting rates increase submicroscopic infections, as described in [28,39,40].

## 5. Conclusions

This study is the first of its kind to be conducted in Mossendjo. The low performance levels of microscopists and the high prevalence of submicroscopic *P. falciparum* infections found in Mossendjo represent a major challenge for malaria control in this remote area, since the management of malaria patients relies on the microscopic detection of parasites. Thus, regular training of routine microscopists alongside the simultaneous use of RDTs and microscopy are needed.

## Figures and Tables

**Table 1:** Sociodemographic and clinical characteristics of patients.

Characteristics	CSIMSJ	HBMSJ	Total *n* (%)
Total number of patients	650	234	884
Age groups *n* (%)			
<5 years	89 (13.69)	16 (6.84)	105 (10.63)
5–14 years	181 (27.85)	25 (10.68)	206 (19.63)
≥15 years	380 (58.46)	193 (82.48)	573 (70.47)
Mean age ± SD	25.96 ± 0.84	32.01 ± 1.26	28.97 ± 2.11
Gender *n* (%)			
Male	282 (43.38)	96 (41.03)	378 (42.21)
Female	368 (56.62)	138 (58.97)	506 (57.80)
Use of mosquito nets *n* (%)			
Yes	604 (92.92)	201 (85.90)	805 (89.41)
No	44 (6.77)	31 (13.28)	75 (10.03)
Unspecified	2 (0.31)	2 (0.85)	4 (0.58)
Treatment 15 days before consultation *n* (%)			
Yes	45 (6.92)	65 (27.78)	110 (17.35)
No	602 (92.62)	168 (71.79)	770 (82.21)
Unspecified	3 (0.46)	1 (0.43)	4 (0.45)
Fever *n* (%)			
Yes	524 (80.62)	123 (52.56)	647 (66.59)
No	123 (18.92)	99 (42.31)	222 (42.31)
Unspecified	3 (0.46)	12 (5.13)	15 (2.80)
Microscopic infections *n* (%)	289 (44.46)	79 (33.76)	368 (39.11)
Mean geometric of parasite density	4845.50 ± 5582.91	4019.79 ± 4251.38	4432.65 ± 4917.45

**Table 2:** Performance of routine microscopy at two health centers in Mossendjo.

Site		Quality Control		Sensitivity % (CI: 95%)	Specificity % (CI: 95%)	PPV % (CI: 95%)	NPV % (CI: 95%)
		Positive	Negative				
CSIMSJ	Positive	143 (22%)	41 (6.31%)	49.5 (43.7–55.2) ^a^	88.6 (85.4–91.9)	77.7 (71.9–82.6)	68.7 (66.0–71.2)
	Negative	146 (22.46%)	320 (49.23%)				
HBMSJ	Positive	26 (11.11%)	32 (13.67%)	32.9 (22.5–43.3) ^b^	79.4 (73.2–85.7)	44.8 (34.3–55.8)	69.9 (66.1–73.4)
	Negative	53 (22.65%)	123 (52.56%)				

^a^: McNemar’s test (*p* < 0.001); ^b^: McNemar’s test (*p* = 0.03); CI: confidence interval: 95%; PPV: positive predictive value; NPV: negative predictive value.

**Table 3:** Identification of plasmodial species between the microscopists of the sites and the experts.

Species	CSIMSJ Microscopists *n* (%)	Experts *n* (%)	HBMSJ Microscopists *n* (%)	Experts *n* (%)	All Sites *n* (%)	Experts *n* (%)
*Pf*	184 (100%)	261 (90.31%)	58 (100%)	70 (88.61%)	242 (100%)	331 (89.94%)
*Pm*	0	15 (5.19%)	0	1 (1.27%)	0	16 (4.35%)
*Po*	0	8 (2.77%)	0	2 (2.53%)	0	10 (2.72%)
*Pf + Pm*	0	4 (1.38%)	0	6 (7.59%)	0	10 (2.72%)
*Pf + Po*	0	1 (0.35%)	0	0	0	1 (0.27%)

Pf: P. falciparum; Pm: P. malariae; Po: P. ovale.

**Table 4:** Relationship between microscopic infection, parasite density, submicroscopic infections, and other characteristics.

Characteristics	Prevalence of Microscopic Infection % (n/N)	*p*-Value	Mean of Log 10 Parasite Density + SD	*p*-Value	Prevalence of Submicroscopic Infections % (n/N)	*p*-Value
Gender						
Male	42.27 (156/378)	0.851	8.65 ± 2.73	0.128	33.33 (126/378)	0.196
Female	41.90 (212/506)		8.23 ± 2.41		37.55 (190/506)	
Use of mosquito nets *						
Yes	39.63 (319/805)	<0.001	8.65 ± 2.32	0.898	37.38 (299/805)	0.005
No	60 (45/75)		8.66 ± 2.33		21.25 (17/75)	
Treatment 15 days before consultation *						
Yes	40.91 (45/110)	0.917	8.88 ± 2.31	0.308	30.91 (34/110)	0.165
No	41.43 (319/770)		8.34 ± 2.59		36.62 (282/770)	

*: Four patients were not recorded; SD: standard deviation.

## Data Availability

The clinical data used to support the results of this study are available upon request from the competent authorities and the corresponding author.
